# Process and outputs from a community codesign workshop on reducing impact of heat exposure on pregnant and postpartum women and newborns in Kilifi, Kenya

**DOI:** 10.3389/fpubh.2023.1146048

**Published:** 2023-08-31

**Authors:** Adelaide Lusambili, Peter Khaemba, Felix Agoi, Martha Oguna, Britt Nakstad, Fiona Scorgie, Veronique Filippi, Jeremy Hess, Nathalie Roos, Mathew Chersich, Sari Kovats, Stanley Luchters

**Affiliations:** ^1^Environmental Health and Governance Center, Leadership and Governance HUB - School of Business, Africa International University, Nairobi, Kenya; ^2^Institute for Human Development, Aga Khan University, Nairobi, Kenya; ^3^Emergency Medicine, Environmental and Occupational Health Sciences, Global Health, University of Washington, Seattle, WA, United States; ^4^Division of Pediatric and Adolescent Medicine, Institute of Clinical Medicine, University of Oslo, Oslo, Norway; ^5^Department of Pediatric and Adolescent Health, University of Botswana, Gaborone, Botswana; ^6^Wits Reproductive Health Institute (WRHI), University of the Witwatersrand, Johannesburg, South Africa; ^7^MARCH, London School of Hygiene & Tropical Medicine, London, United Kingdom; ^8^Department of Medicine, Clinical Epidemiology Division, Karolinska Institute, Stockholm, Sweden; ^9^Centre on Climate Change and Planetary Health, London School of Hygiene & Tropical Medicine, London, United Kingdom

**Keywords:** codesign, climate change, heat exposure, pregnant and postpartum women, newborns

## Abstract

**Background:**

Ambient heat exposure is increasing due to climate change and is known to affect the health of pregnant and postpartum women, and their newborns. Evidence for the effectiveness of interventions to prevent heat health outcomes in east Africa is limited. Codesigning and integrating local-indigenous and conventional knowledge is essential to develop effective adaptation to climate change.

**Methods:**

Following qualitative research on heat impacts in a community in Kilifi, Kenya, we conducted a two-day codesign workshop to inform a set of interventions to reduce the impact of heat exposure on maternal and neonatal health. Participants were drawn from a diverse group of purposively selected influencers, implementers, policy makers, service providers and community members. The key domains of focus for the discussion were: behavioral practices, health facilities and health system factors, home environment, water scarcity, and education and awareness. Following the discussions and group reflections, data was transcribed, coded and emerging intervention priorities ranked based on the likelihood of success, cost effectiveness, implementation feasibility, and sustainability.

**Results:**

Twenty one participants participated in the codesign discussions. Accessibility to water supplies, social behavior-change campaigns, and education were ranked as the top three most sustainable and effective interventions with the highest likelihood of success. Prior planning and contextualizing local set-up, cross-cultural and religious practices and budget considerations are important in increasing the chances of a successful outcome in codesign.

**Conclusion:**

Codesign of interventions on heat exposure with diverse groups of participants is feasible to identify and prioritize adaptation interventions. The codesign workshop was used as an opportunity to build capacity among facilitators and participants as well as to explore interventions to address the impact of heat exposure on pregnant and postpartum women, and newborns. We successfully used the codesign model in co-creating contextualized socio-culturally acceptable interventions to reduce the risk of heat on maternal and neonatal health in the context of climate change. Our interventions can be replicated in other similar areas of Africa and serve as a model for co-designing heat-health adaptation.

## Introduction

High temperature are known to have a range of impacts on human health and wellbeing. It has been estimated that by 2009, heat-related child mortality in sub-Saharan Africa was already double what it would have been without climate change and will double again by 2050 if temperatures exceed 1.5°C of warming (1). High temperature are known to increase the risk of pre-term birth and stillbirth (2–5). With about 15 million babies born prematurely and most in sub-Saharan Africa and Asia (6, 7), pregnant women need to reduce their exposure to ambient heat. High temperatures also have negative effects on the mental health and wellbeing of pregnant women and can interfere with breastfeeding and infant care (8). The health consequences of climate change which are important for pregnant women and neonates also include insufficient food, inadequate supply of water, population displacement and infectious disease outbreaks (5). Adaptation to reduce the impact of climate change hazards on health outcomes is necessary. McMichael and Kovats discussed three adaptive modes: biological (individual level), behavioral (personal level) and social (community level) (9).

The benefits of locally led adaptation in climate change has gained traction (10, 11). Many health interventions tested in clinical settings are not as effective when translated into real-life practice (12). To increase uptake and utility of health interventions, the end-user needs to be meaningfully engaged in codesigning the interventions, either through multiple knowledge generation systems (13) or knowledge co-creation models (14, 15). To the best of our knowledge, there are no published interventions that have been codesigned to reduce the impact of ambient heat exposure on pregnant, postpartum and neonatal health, and particularly in hot arid and semi-arid areas such as Kilifi, Kenya. There is a growing body of evidence on the impacts of heat on these populations but limited research on the interventions to reduce such risks (16–22). It is therefore imperative to codesign contextualized interventions for mitigating effects of extreme heat exposure. In this study, we share our experience and lessons learned from co-developing a set of interventions against the impact of heat exposure on maternal and neonatal health.

## Summary of CHAMNHA study in Kilifi

Our codesign workshop was preceded by a qualitative study conducted in Kilifi County, Kenya, as part of the Climate Heat Maternal and Neonatal Health Africa (CHAMNHA) project. The research was led by the Aga Khan University between February and June 2021 which has worked previously with the local communities. Kilifi County is largely rural (23) with poor infrastructure: water is scarce and of low quality, health facilities are few and far from the community and many homes are grass thatched and unventilated (24). Families are generally large, consisting of 5 to 15 members, and perinatal mortality and morbidity is high (25).

The CHAMNHA research aimed to examine the perceptions toward the effects of high temperatures on women during pregnancy, childbirth and postpartum, and the effects on their neonates. We interviewed community members, including pregnant and postpartum women, key stakeholders from different government ministries, and community members that support women through pregnancy and childbirth. The findings from the qualitative study highlighted the importance of local attitudes to heat and pregnancy in affecting the impacts on pregnant women and newborns (see Table 1) (8, 26).

**Table 1:** Summary of the qualitative study findings.

Domain	Heats direct and indirect pathways
Newborns	High temperatures injure baby’s skins Heat creates discomfort making it difficult for the mother to care and interact with the baby Exclusive and frequent breastfeeding is not possible due to discomfort caused by the heat
Pregnant and postpartum women	Heat leads to delayed antenatal and postnatal care due to the fear of walking in the heat Heat affects livelihoods and women walk longer distances in search of water and firewood Perceived associations with preterm births and C-sections Leads to dehydration in mothers because of working long hours in outdoor heat. Walking in the heat leads to increased hemorrhage in postpartum periods and compromised hygiene.
Home and health facility environment	Health facilities are hot, and lack cooling spaces making them unconducive during labor and delivery Home environments are unventilated and dependency on solid fuel for cooking leads to indoor air pollution Women and their babies have little protection against heat in the home and health facilities
Outside environment	Depleted water sources increase workload for women in search of water There is little shading in the homes and health facilities to provide cooling for women and babies The community lack knowledge on the link between environment and climate change

## Codesign process

The codesign workshop was conducted after a series of meetings and discussions with a group of multidisciplinary professionals from social behavioral, environmental and health sciences, including clinicians in maternal and neonatal health. The findings from Table 1 informed the discussions.

### Step 1: participants sampling and recruitment

Successful collaboration and design require representation of a diverse range of people at different levels of the community, including key influencers, implementers, and end users (27, 28). To have an accurate, representative view of the community, we incorporated diverse participants, including direct beneficiaries or the target group for the intervention (pregnant and postpartum women) and representatives from key ministries that implement and deliver services (ministries of Water, Environment, Housing, Education, Health, Infrastructure and the Department of Meteorology). We also included community leaders and influencers such as religious leaders, local chiefs, community health unit heads and community health volunteers. Women’s household support systems, such as their male spouses and mother-in laws also participated in the codesign workshop (Table 2).

**Table 2:** Codesign participants.

GROUP 1: (*n* = 6)	GROUP 2: (*n* = 7)	GROUP 3: (*n* = 8)
Ministry of Gender Meteorologist Infrastructure Environment Ministry of Health (MOH/county office) Ministry of Housing Ministry of water	Religious leader Chiefs *n* = 2 Non-government organization Health Facility Manager Midwife Male partner/spouse	A community health volunteer (CHV) Postpartum women *n* = 2 Pregnant woman Traditional Birth Attendant *n* = 2 Mothers-in-law *n* = 2

De Freitas and Martin observed the importance of being sensitive to power differentials when working in marginalized communities such as Kilifi (29). They also impressed on the need to provide creative approaches to ensure safe environments for conversations without necessarily increasing tokenism. The project researchers worked with local facilitators and consulted with community health volunteers in Kilifi to ensure that participants were assigned in groups where they could feel comfortable as illustrated in Table 2. These groups were structured to reflect sensitivity to power differentials (gender roles) and ensure a safe and inclusive environment to discuss issues at hand. For instance, female Community Health Volunteers (CHVs), pregnant and postpartum women, mothers in law and traditional birth attendants (TBAs) (group 3) were put in a separate group as it was deemed inappropriate to mix them with representatives from government ministries (group 1), or community leaders (group 2) due to power dynamics, also amplified by language barriers.

Group discussions were structured to focus on topics that could be discussed easily among the group members. For instance, members who were mainly from government ministries (group 1) were tasked with the responsibility of discussing and suggesting interventions to key issues at policy levels, such as improvements in the home/built environment, water scarcity, reforestation, and the shading of communities.

Both group 2 and 3 were tasked to discuss issues at meso-and micro-levels, such as challenges in the health systems that may be detrimental to maternal and neonatal health in periods of high temperatures. These topics had been discussed and decided by the CHAMNHA consortium members following a series of meetings during data analysis (Table 3).

**Table 3:** Codesign discussion topics.

Domain	Qualitative findings	Question	By whom and what?
Behavioral practices	Women refuse to drink water during pregnancy and postpartum periods due to social cultural beliefs around drinking water and their perceived effect dirty and contaminated water had on the unborn child. Pregnant mothers perform heavy workloads in the heat which leads to exhaustion. Mothers do not breastfeed their babies enough due to low milk production and discomfort caused by the heat. Mothers spent many hours away from the baby performing heavy workload in the heat. This is detrimental to both their health and that of the baby. Mothers continue to layer their babies heavily when it is hot due to perceived belief that it protects the baby from being seen by evil people. Women spent many hours with their babies in indoor smoke polluted and unventilated homes. Women do not use mosquito nets when it is hot as it causes discomfort.	What can community do to combat harmful behaviors during heat?	For the suggestions you have given, who should lead?
Health system factors	Health facilities are hot; lack cooling spaces and air conditions undermining the delivery of care during labor, delivery and for the newborn. Distances to health facilities is far and women defer antenatal and postnatal care when it is hot.	What can be done in the facilities against heat?	What challenges exist for implementing these interventions?
Home environment	Unventilated houses exacerbate indoor air pollution from dust and cooking smoke, worsening the heat burden for mothers and babies. There is lack of greening around the homes and pregnant and postpartum mothers have nowhere to escape when it is hot.	What can be done to help women and their newborns in their homes?	What opportunity exists for implementing these interventions?
Water scarcity	Water sources have dried up due to drought, and pregnant, postpartum women walk long distances with their babies in the heat in search of water increasing heat risks. Available water is dirty and contaminated.	What can the community do to ensure that there is sufficient water?	How sustainable are these interventions?
Education and awareness	Communities lack education and understanding on the health effects of heat on mothers and babies. There is need to educate communities on the link between reforestation and improvements in health, such as the importance of shading in lowering ambient temperatures and reducing risks of heat stress.	What interventions and approaches are needed to ensure that the community is educated and made aware on the risks of high temperatures?	Who should be in charge and what should they do to achieve this? (process)

### Step 2: training of facilitators

A total of six facilitators (two females and four males) underwent training prior to the workshop, conducted by the social scientists assigned to the study. The facilitators included individuals who had been part of the formative research (*n* = 2), while the rest (*n* = 4) had extensive experience working in the target community. The training included a review of the CHAMNHA study objectives, findings, content for the workshop and consent process. During the training, we used PowerPoint presentations and role playing within each group to ensure that the facilitators were conversant with the scheduled programs, processes, and content. We also discussed gender and power dynamics, professional roles, identities, and language barriers.

### Step 3: codesign venue and activity design logistics

The codesign workshop was conducted face to face in June 2021 over 2 days. Recognizing vulnerabilities and being aware of the potential barriers to participation, the CHAMNHA team had reached out to our participants before the workshop and discussed the codesign objectives and compensation. Considering that rural Kilifi is geographically sparse with limited public transportation means, the CHAMNHA team provided all the participants with transport. This was to facilitate them to come to the hotel, where they had a full board accommodation for the 2 days. Since the codesign workshop was conducted during Covid-19, there was limited travel and some CHAMNHA members (AL, FS, BN, SL) joined the presentations and discussions sessions via the Zoom platform.

### Step 4: tasks and activities

At the start of the workshop, all the participants were asked to review the qualitative study findings (Table 1), after which they broke out into different rooms in small groups (Table 2) for discussions. Each group had a facilitator and a note taker. Firstly, group facilitators introduced themselves and explained the objectives of the codesign, the group topic of focus and the expected outcomes. Secondly, participants in the small groups introduced themselves to each other and described their roles in the community. Thirdly, facilitators explained methods of participatory design, processes, and active consenting to grant permission for them to record all interactions by photos, audio, and observation notes for further processing. Fourthly, each group and trained facilitator were allocated specific topics to discuss (Table 3) and guided questions to ask the groups. For example, after introducing the group topics, facilitators opened the discussions by asking the group to share their views on what the community can do to help women and their newborns in periods of high temperatures during pregnancy, labor, delivery and postpartum. In addition, participants were asked to discuss the challenges and opportunities that exist in implementing the interventions they had suggested. Lastly, the groups were asked to reflect on the suggested interventions based on their ‘likelihood of success’, ‘cost effectiveness’, ‘effectiveness’ and ‘sustainability’ design characteristics.

Toward the end of the first day, all the groups met together for 15 min to reflect on the day’s activities and explain the program for the second day. Table 3 summarizes the main issues that were deliberated by the groups during the codesign workshop. These topics were discussed and agreed in advance by CHAMNHA consortium after analysis of the qualitative research and a series of meetings.

### Step 5: group presentations

On the second day, all groups met in the conference hall and each group presented their suggested interventions. The presentation focused on the priority interventions, possible challenges, and opportunities that exist in this community for successful intervention. The presentation was audio-recorded and thematically analyzed by researchers. As summarized in Table 4, interventions to improve water systems, behavior change, and education were mentioned by all the groups.

**Table 4:** Summary of group presentations: interventions, challenges, and opportunities.

Group	Interventions	Challenges	Opportunities
Group 1, 2 and 3	**Water** To upgrade water dams and boreholes in the homes and health facilities	Financially expensive and lack of political will at both the local and county office.	There is local manpower and an already existing water infrastructure which can be improved.
Group 1,2 and 3	**Social behavior change campaign** The county office to distribute free safe drinking water to pregnant and post-partum women. CHVs and women support systems to encourage women to use mosquito nets frequently, to boil and drink water during pregnancy and postpartum periods. Key community influences such as the chiefs and CHVs to sensitize women to stop harmful behaviors that endangers the baby, e.g., heavy layering and mothers walking with the newborn in the heat	Social cultural beliefs and lack of alternative resources.	The campaign can leverage on the existing community networks of health care workers (HCWs) and CHVs to disseminate messages in the communities.
Group 1	**Planting trees** Local leaders such as the Chiefs and ministry officials to put a strategy in place to educate communities on the link between trees and environment. Ministry of forestry to provide drought resistant seedlings to the community and encourage greening around the health facilities and homes.	Possibility of pests and disease infestations and community ignorance.	The initiative can leverage on the existing initiatives, e.g., adopt a tree and green school initiatives.
Group 1,2 and 3	**Education** Ministry of health, gender, housing environment to work together to develop a strategy to educate the community on the health risks of heat exposure, constructing ventilated homes and cooling spaces in the health facilities.	Community members may not turn out because heat is normalized in these settings	There is a strong multisectoral support from CHVs, HCWs and local leaders
Group 1	**Built environment** The urgent need to construct health facilities with cooling spaces, build health facilities closer to homes as well as improve home environments	Financial barriers and long process in changing the housing policies.	Availability of land to expand housing and locally available construction materials
Group 2 and 3	**Family support** Initiate educational campaign for family members (mothers-in-law, male partners and communities) to support pregnant and postpartum women with daily activities (e.g., farming, fetching water).	Family support system competing tasks and social cultural factors	Male community health volunteers male spouses can be used as a role model to sensitize communities and shifts some of the social cultural behaviors that are barriers

### Step 6: participants’ reflections

Following the group presentations, participants were given the opportunity to reflect and share their thoughts in the light of all the presentations. Reflections from participants shed light on the interventions they considered as a priority.

Overall, the participants acknowledged that there was an urgent need for interventions targeting the whole community to create awareness and educate them on the effects of heat exposure on maternal and newborn health. Education could incorporate behavior change campaigns to shift some of the socio-cultural practices that are harmful to the mother and newborn in periods of high temperatures. These behaviors, as our research found, include: 1. Some women do not drink enough water or refuse to drink water during pregnancy and postpartum periods. In part this is because, generally the water in these settings is scarce and available water is dirty and contaminated which may require women to boil before drinking. In addition, social cultural beliefs among some clans that “water harms the unborn child or freezes breast milk” may discourage some women. 2. Heavy layering of neonates and babies in the first six months due to social cultural belief that leaving the babies skin lightly covered attracts spells and jinx from witchdoctors. 3. Women performing outdoor activities of daily livelihood in the heat with newborns strapped on their backs because the culture expects them to start working immediately after delivery.

Further, the participants argued for the need to have a community campaign not only to sensitize about the heat related health risks, but also to sensitize family members to support pregnant and postpartum women during periods of high temperatures.

Dealing with water scarcity was discussed as the key intervention that can reduce the discomfort of pregnant and postpartum women both at home and in the health facilities during labor and birth. Kilifian women walk with their new-borns strapped on their back for over one hour in the heat in search for water. Many health facilities do not have water and pregnant women are required to carry their own water from home to the health center during labor and delivery. Besides, the need to expand cooling spaces in the health facilities and in the communities was discussed as a priority area of intervention. Participants noted that health facilities could be constructed closer to the homes and the community can be provided with seedlings to plant trees with the aim of improving shading.

On the contrary, there were some interventions that were suggested during the reflection time that most of the participants considered either not appropriate or not a priority in this context. For example, painting the roofs white was seen as inappropriate, as many of the houses in rural Kilifi are built from mud and require no painting. In the same vein, the nature of the houses cannot sustain rain harvesting due to grass thatched roofs. Providing air conditioning fans in the homes to assist pregnant and postpartum women when the temperatures are high was considered unsustainable as most of the homes do not have electricity and even for the few homes that have electricity, this solution would be too expensive for the locals to afford as energy bills were reported to be high.

### Step 7: ranking

Following the participants reflections and group presentations, the research team summarized the main priorities and invited the participants to do rankings based on the four design characteristics: the likelihood of success, cost effectiveness, effectiveness of the interventions, and sustainability. The ranking was tabulated, and results announced as in Table 5. Based on the rankings, accessibility to water, social behavior change campaign and education were ranked top three interventions for reducing the impact of climate change-related heat exposure on pregnant and postpartum women, and newborns.

**Table 5:** Ranking.

* 1 is the highest rank (I.e., “the best) and 5 is the lowest (the “worst”)				
Ranking	Likelihood of success	Cost Effectiveness	Effectiveness	Sustainability
1	Water	Education	Water	Water
2	Education	Social behavior change campaign	Social behavior change campaign	Social behavior change campaign
3	Social behavior change campaign	Family support	Education	Education
4	Family support	Water	Family support	Family support
5	Construct health facilities	Construct health facilities	Construct health facilities	Construct health facilities

## Lessons learned

### Capacity building

Research on the association between heat exposure and overall health risks is an unexplored area in this Kenyan context. Our codesign workshop was used as an opportunity to build capacity among facilitators and participants. Our participants confessed that they were not aware of health-related risks of heat exposure, and that our presentation of the research findings was informative, as it provided insights on experiences, they had been observing in Kilifi County. Building capacity among key community influencers, such as chiefs, religious leaders, community health volunteers and male spouses, was suggested to be of greater impact as these individuals would share the information in various local communities and decisive making authorities. Creating such awareness would help community to begin finding solutions to protect themselves and, particularly, those who are vulnerable.

### Segmentation of the groups and facilitators contributed to the richness of the interventions

Our codesign had diverse participants drawn from different levels including the beneficiaries (micro), community influencers (meso) and policy makers at the various ministries (macro). Various topics were also discussed based on the individuals’ roles in the society, such as allocating individuals to groups where they felt comfortable to share their views was key to finding beneficial solutions. During the plenary discussions and reflections, the priority interventions were well in line with all the participants. Participants felt that both water and social behavioral change campaigns should be a priority intervention that can be sustainable, while on the other hand they were very clear on interventions considered not fit for this context. For example, where interventions such as installing air fans and painting roofs white have been used elsewhere and found to have a positive effect (30), in this context, it was not deemed appropriate. This revelation shows that, although the effect of heat exposure across the communities may be the same, interventions may vary based on the context they will adapt to and there is no “one size-fits-all” interventions. As extreme weather continues to ravage many communities in sub-Saharan Africa, it is important that different players and stakeholders at different levels be consulted in making decisions they consider appropriate in their own communities.

### Training facilitators

A lesson learned from this codesign was the importance of training the facilitators in advance and giving them opportunity to reflect on possible challenges before the codesign workshop. Our group facilitators underwent a thorough two-day training, and one of the foreseen challenges was the possibility of being distracted by participants diverting discussions to their current economic challenges such as unemployment and a lack of livelihood. We felt that sufficiently training facilitators and giving them possible dips on how to negotiate with participants when such emotive questions arose was important in enhancing rapport building with the participants.

### Context specific challenges

CHAMNHA researchers faced various challenges that are key for future codesign workshops in similar settings. The rural communities in Kilifi County are geographically dispersed, with limited public transport. For logistical purposes, we conducted our codesign workshop for 2 days in a hotel where all the participants stayed. The consequence of this was that, recruiting pregnant and postpartum women who had to spend 2 days at the hotel was made by cultural barriers as their male spouses and partners did not consent, a problem experienced by women in these communities. The women who accepted to attend the workshop had to be accompanied with their mothers-in-law. Postpartum women attending the conference came with infants and other young children in their households. This led to additional expenditure for the project. It must be noted that in the Kilifi rural community, male spouses/partners and mothers-in-law feel insecure having young wives spend the nights outside without their presence. Future codesign workshops should invite pregnant and postpartum women, but they should allocate a budget able to cover the need for spouses, childcare and other influential household member such as mothers-in-law who may want to attend and provide protection for their daughters-in law. Alternatively, codesign should be conducted in the communities close to where women live.

There were many interruptions during the session. For religious considerations, many of the participants had to leave the meetings to pray, while some mothers had to be given time to breastfeed. These considerations must be factored in future codesign. Moreover, the codesign was conducted when temperatures were high in Kilifi and participants suffered from symptoms of heat exhaustion.

Lastly, we noticed that during our community entry when we involved and informed different ministries, key influencers and community members about our research, they did not seem to understand the objectives of our community-related research as they did not fully appreciate that ambient heat may have adverse effects on the health of pregnant and postpartum mothers and newborn children. This implied that the facilitators had to spend much time explaining our research to participants before the actual discussions could begin.

## Discussion

The codesign workshop was developed following a series of meetings and reflections where CHAMNHA consortium members closely interrogated and discussed data from the formative research. The process also entailed working closely with field researchers and community health volunteers to further refine some of the findings and areas of interventions that were discussed during the workshop. CHAMNHA successfully utilized the codesign model to identify and prioritize contextualized socio-culturally acceptable interventions to reduce the impact of climate heat exposure hazards on maternal and neonatal health in Kilifi County. Fruitful discussions were carried out and useful conclusions were made. Accessibility to water, social behavior change campaigns and education were ranked the top three sustainable interventions as these were evaluated as effective with the highest likelihood of success.

The codesigned interventions aligned with behavioral and social adaptive models as well as the primary, secondary and tertiary prevention categories (9). On primary prevention, change in social norms where family support would ensure that neonates are wrapped in fewer layers of clothing and staying in the coolest possible places as women carry out their outdoor daily livelihood chores. Additionally, planting trees for shade, cooling spaces, ventilation and insulation in houses or health facilities would reduce the risk of exposure to excess heat. These interventions need significant awareness and knowledge sharing and, therefore could explain why social behavior change campaigns and education ranked among the top recommended interventions. Creating awareness through public education on practices such as avoiding heat and rehydration to replenish lost body fluids have been suggested to reduce illness related to thermal stress (9, 30).

Secondary prevention measures included making water more accessible, which would ensure that women do not walk long distances in search of water while exposing themselves to unbearable heat and dehydration for poor maternal and neonatal health. The availability of safe drinking water will help women and children stay rehydrated and encourage them to drink more water during pregnancy, and improve hygienic practices such as hand washing, food/fruits cleaning and general sanitation.

On tertiary prevention, the construction of additional health facilities would increase accessibility to the healthcare system for curative interventions against the impact of heat exposure. However, this was ranked the least important high across the four designed characteristics. Nevertheless, if health facilities were closer to the population at the community level, then women would commute shorter distances in search of health care, reducing exposure to excess heat. Additionally, this will improve health seeking behavior during the perinatal period that would improve overall health outcomes, as part of primary and secondary prevention strategies against the impact of heat exposures.

It was surprising to see family support ranked in the top three on perceived intervention cost-effectiveness, displacing water intervention which ranked as number four. This suggested that the community feels that making safe water available would require significant investment. Indeed, the coastal Kenyan region relies on sparsely distributed freshwater rivers making accessibility to clean, safe fresh water much harder.

Implementation of this codesign navigated several challenges: overcoming community participants’ level of education through a paired-up approach, accommodating socio-cultural and religious norms and traditional practices during the codesign sessions, flexibility in financial planning to meet unexpected costs related to community participations’ facilitation to attend codesign sessions, and stakeholders’ knowledge gaps on targeted communities requiring extensive recruitment mobilization and intensive study sensitization. Therefore, when planning codesign sessions, prior to this contextualizing of local set up, cross-cultural and religious practices and budget considerations would increase the chances of a successful outcome of interventions to promote neonatal and maternal health in very hot settings.

## Conclusion

The link between heat exposure and overall health risks is an unexplored topic in Kilifi, Kenya but is of increasing importance under climate change even globally. Our experience shows that codesign interventions on heat exposure with diverse stakeholder process yielded some unexpected findings. The codesign workshop was successfully utilized as an opportunity to build capacity among facilitators and participants and explore interventions to address the impact of heat exposure on pregnant, and postpartum women and newborns. Accessibility to water, social behavior change campaign and education were ranked as the top three interventions with the highest likelihood of success. Family support was also ranked fourth and could easily be integrated with social and behavior change campaigns. The codesign approach provides unique opportunities for developing interventions with various stakeholders to address the impact of climate change-related heat exposure on pregnant, postpartum women and newborns. Future studies should test the effectiveness of these interventions.

## Data availability statement

The raw data supporting the conclusions of this article will be made available by the authors, without undue reservation.

## Ethics statement

The studies involving humans were approved by Aga Khan University Ethics Committee ref. 2020/IERC-94 (v2) and National Commission for Science and Technology and Innovation Ref BAHAMAS ABS/P/20/7568, London School of Hygiene and Tropical Medicine Ref: 22685 and from Kilifi County Office ref. DOM/KLF/RESCH/vol.1/66. The studies were conducted in accordance with the local legislation and institutional requirements. Written informed consent for participation in this study was provided by the participants’ legal guardians/next of kin.

## Author contributions

AL wrote the first draft. All authors reviewed the manuscript, contributed to the article, and approved the submitted version.

## Funding

The CHAMNHA was funded by the Natural Environment Research Council (grant numbers NE/T013613/1 and NE/T01363X/1), the Research Council of Norway (grant number 312601) and The Swedish Research Council for Health, Working Life and Welfare in collaboration with the Swedish Research Council (Forte) (grant number 2019-01570) and the National Science Foundation (NSF) (grant number ICER-2028598), coordinated through a Belmont Forum partnership.

## Conflict of interest

The authors declare that the research was conducted in the absence of any commercial or financial relationships that could be construed as a potential conflict of interest.

## Publisher’s note

All claims expressed in this article are solely those of the authors and do not necessarily represent those of their affiliated organizations, or those of the publisher, the editors and the reviewers. Any product that may be evaluated in this article, or claim that may be made by its manufacturer, is not guaranteed or endorsed by the publisher.
